# National Society for Employment of Epileptics

**Published:** 1899-06-17

**Authors:** 


### NATIONAL SOCIETY FOR EMPLOYMENT OF EPILEPTICS.

The annual meeting of governors was held on Monday at the offices of the society, 12, Buckingham Street, Strand, the chair being taken by Mr. E. Montefiore Nicholls. The Chairman, in his introductory speech, referred to the forthcoming opening by the Duke and Duchess of York of four new homes at the colony established by the society at Chalfont. Two of these homes were intended for children, and the committee looked to this extension of the work with the most confident anticipations of good results. The progress of the society during its brief existence had been almost without a parallel amongst charitable institutions. According to the report of the honorary medical staff, "a marked improvement has been noted in the majority of the cases, both as regards the general state of health and the disease." In regard to young people from fifteen to eighteen years of age "without exception these youths have done remarkably well, while as to the female patients the effects have been very good indeed."

